# Complications from Surgeries Related to Ovarian Cancer Screening

**DOI:** 10.3390/diagnostics7010016

**Published:** 2017-03-08

**Authors:** Lauren A. Baldwin, Edward J. Pavlik, Emma Ueland, Hannah E. Brown, Kelsey M. Ladd, Bin Huang, Christopher P. DeSimone, John R. van Nagell, Frederick R. Ueland, Rachel W. Miller

**Affiliations:** Division of Gynecologic Oncology, Department of Obstetrics and Gynecology, The University of Kentucky Chandler Medical Center and the Markey Cancer Center, 800 Rose Street, Lexington, KY 40536-0293, USA; labald1@uky.edu (L.A.B.); Epaul1@uky.edu (E.J.P.); emmaueland_2017@depauw.edu (E.U.); hannah.e.brown@duke.edu (H.E.B.); Kelsey.ladd@uky.edu (K.M.L.); bhuang@kcr.uky.edu (B.H.); cpdesi00@uky.edu (C.P.D.); jrvann2@email.uky.edu (J.R.v.N.); fuela0@email.uky.edu (F.R.U.)

**Keywords:** ovarian cancer screening, complications, ovary, cancer, screening

## Abstract

The aim of this study was to evaluate complications of surgical intervention for participants in the Kentucky Ovarian Cancer Screening Program and compare results to those of the Prostate, Lung, Colorectal and Ovarian Cancer Screening trial. A retrospective database review included 657 patients who underwent surgery for a positive screen in the Kentucky Ovarian Cancer Screening Program from 1988–2014. Data were abstracted from operative reports, discharge summaries, and office notes for 406 patients. Another 142 patients with incomplete records were interviewed by phone. Complete information was available for 548 patients. Complications were graded using the Clavien–Dindo (C–D) Classification of Surgical Complications and considered minor if assigned Grade I (any deviation from normal course, minor medications) or Grade II (other pharmacological treatment, blood transfusion). C–D Grade III complications (those requiring surgical, endoscopic, or radiologic intervention) and C–D Grade IV complications (those which are life threatening) were considered “major”. Statistical analysis was performed using SAS 9.4 software. Complications were documented in 54/548 (10%) subjects. For women with malignancy, 17/90 (19%) had complications compared to 37/458 (8%) with benign pathology (*p* < 0.003). For non-cancer surgery, obesity was associated with increased complications (*p* = 0.0028). Fifty patients had minor complications classified as C–D Grade II or less. Three of 4 patients with Grade IV complications had malignancy (*p* < 0.0004). In the Prostate, Lung, Colorectal and Ovarian Cancer Screening trial, 212 women had surgery for ovarian malignancy, and 95 had at least one complication (45%). Of the 1080 women with non-cancer surgery, 163 had at least one complication (15%). Compared to the Prostate, Lung, Colorectal and Ovarian Cancer Screening trial, the Kentucky Ovarian Cancer Screening Program had significantly fewer complications from both cancer and non-cancer surgery (*p* < 0.0001 and *p* = 0.002, respectively). Complications resulting from surgery performed as a result of the Kentucky Ovarian Cancer Screening Program were infrequent and significantly fewer than reported in the Prostate, Lung, Colorectal and Ovarian Cancer Screening trial. Complications were mostly minor (93%) and were more common in cancer versus non-cancer surgery.

## 1. Introduction

Ovarian cancer is the most common cause of gynecologic cancer death in the United States with 22,280 new cases and 14,240 deaths from the disease in 2016 [1]. Despite the introduction of targeted therapies, refinements in novel chemotherapy regimens, and advances in surgical techniques, survival outcomes have remained essentially unchanged over time [2]. Most patients with ovarian cancer are diagnosed with advanced stage disease where survival outcomes are poor. Surgical stage at the time of diagnosis remains among the most important prognostic factors for patients with ovarian cancer. Women with Stage I disease, where cancer is confined to one or both ovaries have a 10-year survival rate of 74%, whereas those with Stages II, III, and IV disease have 10-year survival rates of 45%, 21%, and <5%, respectively [3]. Identifying women with early stage disease is difficult since early ovarian cancer does not reliably cause symptoms. A specific symptom profile has been described in patients with ovarian cancer; however, it is most often reported in those with advanced stage disease [4]. Early stage disease rarely demonstrates this symptom profile [5,6].

The key to a successful screening program is the increased detection of early stage disease and subsequent improved survival in the screen-detected cancers. Efforts in ovarian cancer screening have focused on the integration of transvaginal sonography and serum biomarkers, specifically CA 125 [7,8,9,10]. Improved survival from ovarian cancer screening has been reported [11,12,13], especially with regard to screen-detected incident ovarian cancers [9,14]. One large trial (the Prostate, Lung, Colorectal and Ovarian (PLCO) Randomized Controlled Screening Trial: *PLCO trial*) failed to observe improved survival in the intervention (screening) group [15] and reported a surprisingly high false positive rate with 19 women recommended for surgery for every malignancy that was identified [16]. This is in contrast to other screening studies that reported lower false positive rates [9,11,13]. In the PLCO trial, screen positive cases found to be non-malignant at surgery had an unexpectedly high complication rate (15%) [15] and led to announcements that ovarian cancer screening does more harm than good [17]. In comparison, the United Kingdom Controlled Trial on Ovarian Cancer Screening (UKCTOCS trial) reported a surgical complication rate of less than 1% [9].

The present study examines complications in women undergoing surgery as a result of an abnormality detected in the Kentucky Ovarian Cancer Screening Program, an ultrasound-based program that has screened over 40,000 women from 1988 to present. We objectively evaluated the number and type of complications observed in these women using the Clavien–Dindo (C–D) Classification of Surgical Complications [18,19] and compared findings to those reported in the PLCO trial.

## 2. Methods

**Figure 1:**
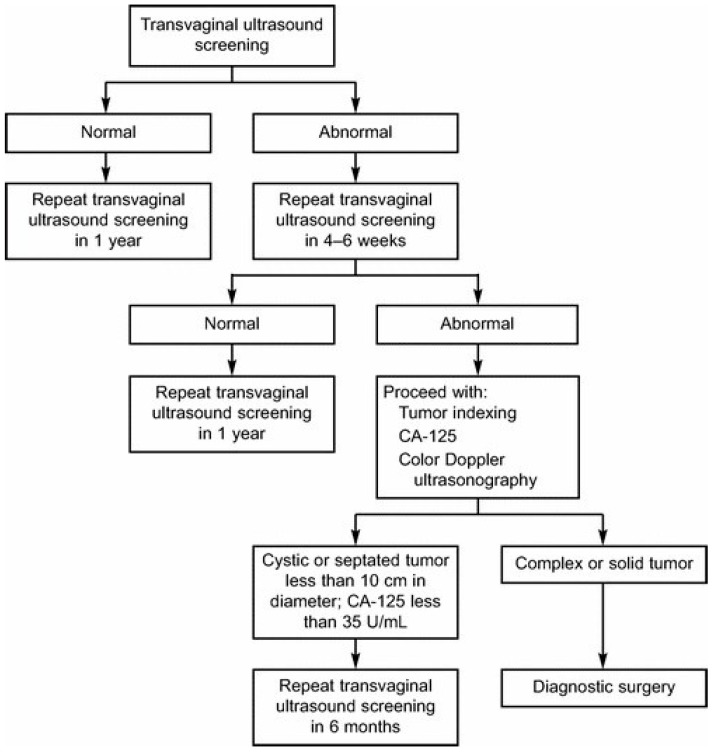
Study algorithm for the Kentucky Ovarian Cancer Screening Program. Reprinted from [12].

**Figure 2:**
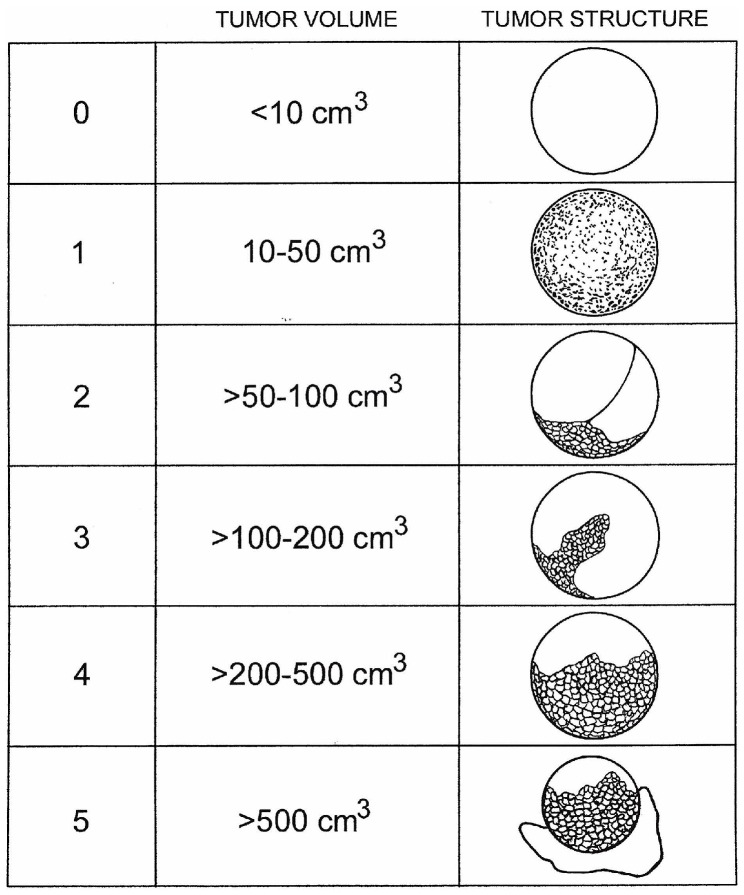
Morphology Index (numeric value 0–10). Reprinted from [12].

The study was approved by the (University of Kentucky Institutional Review Board protocol 88-0021-9F with the most recent renewal on 11 August 2016. Women enrolled in the Kentucky Ovarian Cancer Screening Program from 26 May 1988 to 1 June 2014 were included in the study group (*n* = 41,529). The University of Kentucky Institutional Review Board approved this study. Women were recruited by physician referral, media announcements, and word of mouth. Eligibility criteria included asymptomatic women age 50 years or older without a family history of ovarian cancer, or those 25 years or older with a documented family history of ovarian cancer in at least one first or second-degree relative, and the ability to read and understand the informed consent presented in English. Women under clinical evaluation because of pelvic symptoms, a known ovarian tumor, or a personal history of ovarian cancer were excluded. Women enrolled in the Kentucky Ovarian Cancer Screening Program underwent annual screening with transvaginal sonography. Abnormalities were managed according to the study algorithm (Figure 1), which included increased frequency of screening with transvaginal sonography, assessment of morphology index score, and serum CA 125 (Figure 2). Diagnostic surgical intervention was recommended if results indicated at least moderate risk of malignancy according to the published protocol [20]. Minimally invasive surgical technique was preferred, unless medical issues prohibited this approach. Details of the study algorithm, threshold for intervention, and cancer outcomes have been previously published [12,20].

In the first 26 years of the Kentucky Ovarian Cancer Screening Program, 657 patients underwent surgical intervention for positive screens. Three investigators performed a thorough review of all available medical records including operative reports, discharge summaries, and office notes. Phone interviews were conducted when medical records were incomplete. A complication was defined as any deviation from the normal postoperative course within 60 days of surgery. Complete information was obtained for 548 patients. Physician investigators graded all surgical complications that were identified in these 548 patients according to the C–D Classification of Surgical Complications (Table 1) [18,19]. Complications were considered “minor” if they were C–D Grades I or II. Grade I complications included any minor deviations from a normal postoperative course without the need for pharmacologic intervention. Grade II complications consisted of complications treated pharmacologically. C–D Grade III complications (those requiring surgical, endoscopic, or radiologic intervention) and C–D Grade IV complications (those which are life threatening) were considered “major.”

Descriptive analysis for demographics and clinical factors was performed. We used *χ*^2^ tests to examine associations between complication status (yes and no) and other factors such as age, race, body mass index (BMI), type of surgery, cancer status, and type of hospital where surgery was performed. Multivariate logistic regressions were fitted to evaluate the association between complication status and other factors. The final model included only covariates with a significance level of 0.05 or less. Model goodness of fit, multicollinearity, and interactions were also examined. All analyses were performed using SAS Statistical software version 9.4. All statistical tests were two-sided with a *p*-value ≤0.05 used to identify statistical significance. 

## 3. Results

**Figure 3:**
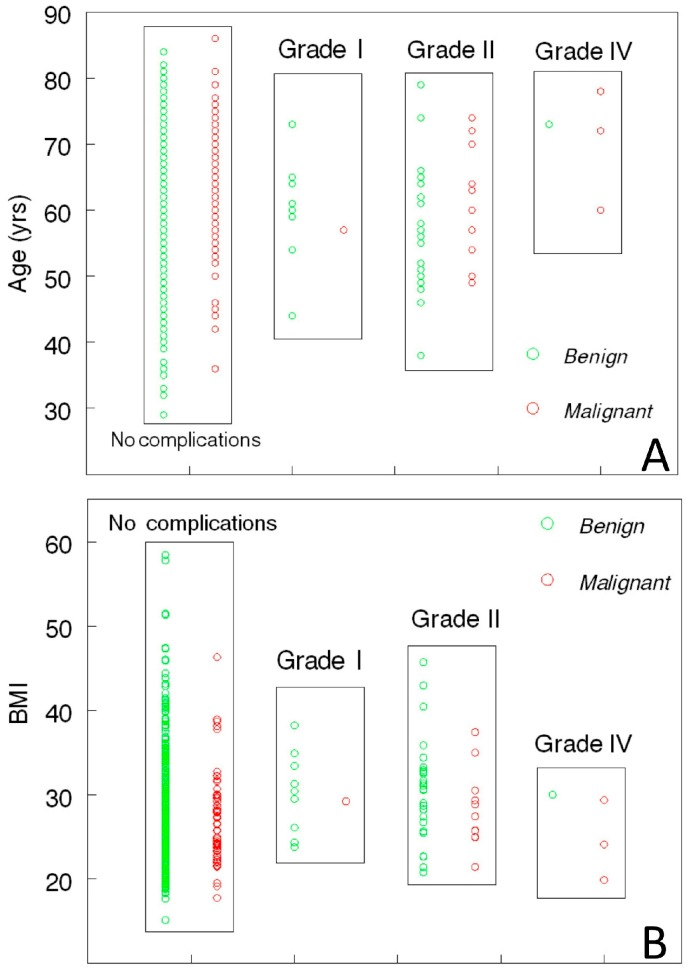
Clavien–Dindo classification of complication relative to age (**A**) and BMI (**B**) in women with benign (**green** circles) and malignant results (**red** circles) at surgery.

Complete clinical information was available on 548 of the 657 patients who underwent surgery for positive screens in the Kentucky Ovarian Cancer Screening Program between the years of 1988–2014. A summary of demographic information is presented in Table 2 and shows that women with and without complications were similar. Complications were documented in 54 of 548 (10%) subjects. Fifty patients (93%) had minor complications classified as C–D Grade II or less, while four had complications categorized as C–D Grade IV. Complication profiles for individuals are shown relative to age and BMI in Figure 3.

**Figure 4:**
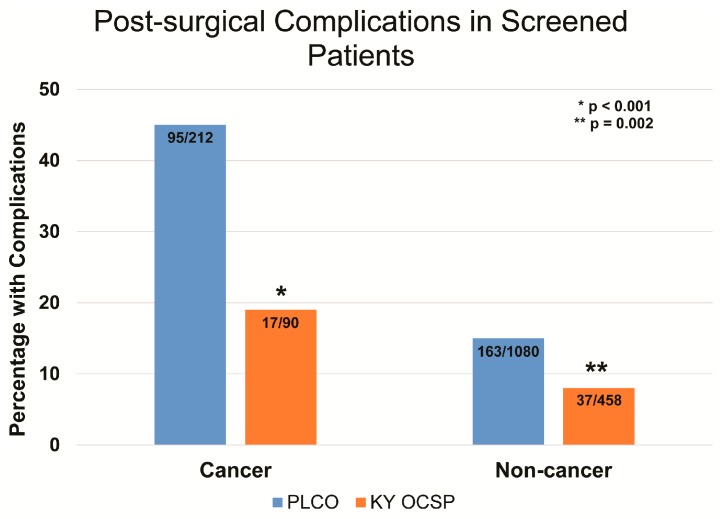
Complications associated with surgery.

Complication rates were compared for surgeries that resulted in the diagnosis of malignancy versus surgery for false positive screens with benign pathology. For women with malignancy, 17 of 90 (19%) had complications compared to 37 of 458 (8%) with benign pathology (*p* < 0.003), Figure 4. Thus, a diagnosis of cancer increased the likelihood of complications with an odds ratio of 2.65. Three of four patients with C–D Grade IV complications had malignancy, while one Grade IV complications occurred in the benign conditions group (*p* < 0.0004). In the PLCO trial, 212 women in the intervention group had surgery for ovarian malignancy, and 95 had at least one complication (45%). Of the 1080 women with surgery with a benign outcome, 163 had at least one complication (15%), yielding an odds ratio of 3 for complications in surgical cancer cases over benign surgical cases. Complication rates from the Kentucky Ovarian Cancer Screening Program were compared with the PLCO trial results. Compared to the PLCO trial, the Kentucky Ovarian Cancer Screening Program had significantly fewer complications from both cancer (*p* < 0.001) and non-cancer surgery (*p* = 0.002) based on chi-square analysis (Figure 4).

Bivariate analysis of complication status versus other clinical variables was performed and obesity was associated with increased incidence of complication, *p* = 0.049 (Table 3). Evaluating clinical variables by cancer status, obesity was not associated with increased complications in surgeries performed for non-cancer pathology, *p* = 0.458 (Table 4). While patients with a cancer diagnosis were significantly older than those with a benign diagnosis, *p* = 0.002 (Table 4), age was not different for those who had complications when compared to those that did not, *p* = 0.463 (Table 3). Other factors evaluated in bivariate analysis did not show significant differences based on complication status.

In multivariate analysis, obesity was determined to be associated with increased risk of complication versus normal weight (OR 3.17, 1.46–6.90). The location where the procedures were performed was also significantly associated with complication risk (OR 1.97, 1.07–3.65) (Table 5).

## 4. Discussion

Ovarian cancer is the second most common gynecologic cancer, but the most common cause of gynecologic cancer death. Most women have advanced disease at the time of their diagnosis, with cancer spread throughout the peritoneal cavity and occasionally into the pleural cavity. Despite aggressive surgery and chemotherapy [21], the five-year overall survival for patients with advanced ovarian cancer is less than 30%. Unfortunately, only about 25% of women present with early stage ovarian cancer, where the five-year overall survival may exceed 80%–90% with appropriate surgical staging and adjuvant therapy. 

Ovarian cancer screening with transvaginal sonography and serum biomarkers has been explored as a means for increasing the number of women diagnosed with early stage disease [7,8,9,10,11,12,13,20]. This shift in stage at diagnosis should result in an improved overall survival as a result of screening. There is a need for ovarian cancer screening because early stage disease rarely produces reliable symptoms. Goff and colleagues reported a symptom profile associated with ovarian cancer [4,22], which included abdominal pain or bloating, pelvic pain, and urinary symptoms present for more than two weeks out of the month and persisting for fewer than 12 months. The effectiveness of a symptom profile is limited as a screening tool because the profile is most useful for identifying advanced stage disease. 

Ovarian cancer screening presents unique challenges that are inherent to the disease itself. First, ovarian cancer has a low incidence with only 22,280 new cases expected in 2016, compared to breast or colorectal cancer in women with 246,660 and 68,830 new cases, respectively [1]. The annual balance of deaths from disease to incident cases for ovarian cancer (0.639) is 3.9 times higher than for breast (0.164) and 1.8 times higher than for colorectal cancer (0.346), indicating that ovarian cancer is a much deadlier disease. This is reflected in the low prevalence of ovarian cancer with an estimated 195,767 women living with the disease in the United States in 2013, relative to colorectal (1,177,556) and breast cancers (3,053,450) [23]. 

A second challenge in ovarian cancer screening is the lack of a thorough understanding of the etiology and natural history of ovarian, primary peritoneal, and fallopian tube cancer. Historically, ovarian cancer was thought to arise from the surface epithelium of the ovary. However, this did not explain normal size ovaries as seen in primary peritoneal cancers. The similarities between serous ovarian and primary peritoneal cancers from the standpoint of genetic mutations, histology, behavior, and response to treatment suggest similar etiologic factors. More recently, investigators have hypothesized that ovarian, primary peritoneal, and fallopian tube cancers originate from serous intraepithelial carcinomas in the fallopian tube [24,25,26,27,28,29,30,31,32,33]. If this is the case, then screening for abnormalities of the ovary with transvaginal sonography will prove futile because the early abnormalities exist in the fallopian tube. This model is founded on the presence of microscopic disease that is below the resolution of biomarkers and ultrasonography, and consequently implies that these screening tools cannot be effective. However, the discovery of Stage I cancers in several screening studies indicates that biomarker and ultrasonography screening modalities are sufficiently effective in detecting ovarian cancer early enough to decrease mortality and increase survival [9,10,11,12]. Thus, cases that have progressed beyond microscopic disease in the distal fallopian tube can be detected by biomarker and ultrasonography screening often enough to achieve a favorable prognosis for extending survival. 

In the present report, we evaluate the complications related to surgery for a positive ovarian cancer screen. In other cancers, such as breast, colon, and cervical, a diagnostic biopsy is performed to determine the presence or absence of malignancy. Percutaneous or transvaginal biopsy of ovarian abnormalities is not recommended because of concern for “seeding” the needle track in the case of malignancy, or for rupturing a malignant tumor, resulting in potentially worse outcomes. Given the aggressive nature of ovarian cancer, these two possibilities could impact the need for adjuvant treatment, or increase the risk of recurrence in early stage disease. As a result, patients with a positive screen indicating a moderate to high risk of malignancy are offered definitive surgery for diagnosis. In most cases, removal of an ovary or ovaries because of an abnormality detected on ovarian cancer screening can be accomplished using a minimally invasive technique, but there are situations when this is not medically recommended. Surgical exploration for a positive screen introduces the possibility of intervention for benign or false positive ovarian abnormalities. The combination of a high percentage of surgeries for women without a malignancy in the PLCO trial (1 malignancy for every 19 surgeries) coupled with a high complication rate [15] led to published statements that screening is harmful [17].

In conclusion, little has been published regarding the nature of the complications reported from surgeries resulting from ovarian cancer screening. In this investigation, we report a low complication rate, with 93% classified as minor. Similarly, the UKCTOCS trial reported a very low complication rate of less than one percent in both screening groups [9]. The procedures of the PLCO trial were to notify the referring physician that a screen was abnormal, but not to make recommendations on whether surgery should be performed or by whom. It is possible that the high complication rates reported in the PLCO trial [15] are related to the recent recognition that better outcomes are achieved when ovarian cancer is treated by specialists at high volume hospitals [34,35,36,37,38,39], and this benefit may particularly apply to early stage ovarian cancers [40]. Ultimately, the methods used to decide who went to surgery and who would perform the operation may best explain the high false positive rates and high complication rates observed in the PLCO trial.

## Figures and Tables

**Table 1:** Classification of Surgical Complications. Modified from [19].

C–D Grades	Definition
Grade I	Any deviation from normal postoperative course without the need for pharmacological treatment or surgical, endoscopic and radiological interventions. Acceptable therapeutic regimens are: drugs as antiemetics, antipyretics, analgetics, diuretics, and electrolytes and physiotherapy.
Grade II	Requiring pharmacological treatment with drugs other thanthan such allowed for Grade I complications.
Grade III	Requiring surgical, endoscopic or radiologic intervention.
Grade III-a	Intervention not under general anesthesia.
Grade III-b	Intervention under general anesthesia.
Grade IV	Life threatening complications (including CNS complications) ^‡^ requiring IC/ICU management.
Grade IV-a	Single organ dysfunction (including dialysis).
Grade IV-b	Multi organ dysfunction.
Grade V	Death of patient.
Suffix “d”	If the patient suffers from a complication at the time of discharge (see examples in Appendix B, http://Links.Lww.com/SLA/A3), the suffix “d” (for “disability”) is added to the respective grade of complication. This label indicates the need for a follow-up to fully evaluate the complication.

^‡^ Brain hemorrhage, ischemic stroke, subarachnoid bleeding, but excluding transient ischemic attacks (TIA); IC: intermediate care; ICU: intensive care unit (www.surgicalcomplications.info).

**Table 2:** Demographics of the group studied.

Variable	No Complications	Complications	Excluded
	*N* = 494	*N* = 54	*N* = 109
Age	59.7, 59 (29–86)	59.6, 59 (38–79)	59.6, 60 (36–84)
Weight	163.6, 158.5 (80–368)	173.7, 170 (121–274)	159.2, 150 (101–250)
Height	64.6, 64.5 (55–71)	64.4, 65 (60–70)	64.5, 64 (57–72)
BMI	27.6, 26.6 (15.1–58.4)	29.4, 29.2 (19.9–45.7)	26.9, 25.8 (18–43.9)
Family history of:			
Ovarian cancer	132 (26.7%)	15 (27.8%)	36 (33%)
Breast cancer	27 (50%)	217 (43.9%)	46 (42.2%)
Breast cancer personal history	8 (14.8%)	39 (7.9%)	8 (7.3%)
Colon cancer	128 (25.9%)	11 (20.3%)	46 (24.7%)
Colon cancer personal history	3 (0.6%)	0 (0%)	1 (0.9%)
No history of hormone replacement therapy	372 (75.3%)	43 (79.6%)	65 (59.6%)
History of hormone replacement therapy	122 (24.7%)	11 (20.4%)	38 (34.9%)
Menopausal Status			
Premenopausal	73 (14.8%)	6 (11.1%)	18 (16.5%)
Perimenopausal	18 (3.6%)	0	7 (6.4%)
Postmenopausal	403 (81.6%)	48 (88.9%)	84 (77.1%)
Any symptoms	254 (51%)	30 (55.5%)	51 (46.8%)
Ovarian cancer symptoms *	27 (14.8%)	4 (7.4%)	5 (4.6%)
Other symptoms **	248 (50.2%)	30 (55.6%	48 (44%)

Mean, median (range) * Women reporting pelvic or abdominal pain, being unable to eat normally, feeling full quickly, feeling abdominal bloating or increased abdominal size presenting for >12 days per month with an onset in less than the last 12 months. ** Women reporting back pain, indigestion, nausea, vomiting, weight loss, urinary urgency, frequent urination, constipation, menstrual irregularities, bleeding after menopause, pain during intercourse, fatigue, leg swelling, difficulty breathing. Any symptoms: any symptom included under ovarian cancer symptoms or other symptoms without regard to frequency of duration.

**Table 3:** Associations between complications and other factors.

Variables	Complications		No Complications		*p*-Value
	*N*	%	*N*	%	
Age					0.463
<50	7	13.0	75	15.2	
50–64	32	59.3	257	52.0	
65–74	10	18.5	121	24.5	
75+	3	5.6	35	7.1	
Unknown	2	3.7	6	1.2	
Weight					0.049
Under-weight	0	0.0	5	1.0	
Normal	11	20.4	185	37.4	
Over-weight	20	37.0	175	35.4	
Obese	20	37.0	108	21.9	
Extreme obesity	3	5.6	21	4.3	

**Table 4:** Patient characteristics by cancer status.

Variables	Cancer		Non-Cancer		*p*-Value
	*N*	%	*N*	%	
Age					0.002
<50	6	6.7	76	16.6	
50–64	38	42.2	251	54.8	
65–74	33	36.7	98	21.4	
75+	11	12.2	27	5.9	
Unknown	2	2.2	6	1.3	
C–D Grade	N	%	N	%	<0.001
None	73	81.1	421	92.0	
Minor	14	15.6	36	7.9	
Severe	3	3.3	1	0.2	
Weight					0.458
Under-weight	1	1.1	4	0.9	
Normal	37	41.1	159	34.7	
Over-weight	31	34.4	164	35.8	
Obese	20	22.2	108	23.6	
Extreme obesity	1	1.1	23	5.0	

**Table 5:** Odds ratio estimates.

Effect	Odds Ratio	95% Confidence Limits
Age		
Unknown vs. 50–64	4.75	(0.86–26.19)
<50 vs. 50–64	0.76	(0.32–1.81)
75+ vs. 50–64	0.77	(0.22–2.71)
65–74 vs. 50–64	0.69	(0.33–1.48)
Weight		
Overweight vs. Underweight/Normal	2.06	(0.95–4.49)
Obese vs. Underweight/Normal	3.17	(1.46–6.90)
Location		
UK vs. Non-UK	1.97	(1.07–3.65)
